# Effect of mesh fixation in incisional hernia repair using the open sublay technique: results from the herniamed-registry

**DOI:** 10.1007/s00423-025-03714-8

**Published:** 2025-04-23

**Authors:** P. Schelbert, RN. Vuille-dit-Bille, F. Köckerling, D. Adolf, R.F. Staerkle

**Affiliations:** 1 University of Lucerne, Lucerne, Switzerland; 2 Department of Pediatric Surgery, University Children’s Hospital of Basel, Basel, Switzerland; 3 Hernia Center, Vivantes Humbold Hospital, Academic Teaching Hospital of Charité University Medicine, Berlin, Germany; 4 StatConsult GmbH, Magdeburg, Germany; 5 Ventravis– Practice for Abdominal Surgery, Dorfplatz 1, Cham, 6330 Switzerland

**Keywords:** Incisional hernia repair, Mesh fixation, Complications, Risk factors

## Abstract

**Purpose:**

Incisional hernias reflect a common complication after abdominal surgery. Main treatment consists of defect closure and mesh insertion using the sublay method. The aim of the present study was to assess the association of mesh fixation to patients’ outcome.

**Methods:**

Using the Herniamed registry, data from 13’452 incisional hernia repairs were analyzed retrospectively. Three groups of patients were compared: those with mesh fixation (*n* = 9’986), those with self-fixing meshes (*n* = 2’725), and those without mesh fixation (*n* = 741). Postoperative complications, recurrence and postoperative pain scores were assessed over a follow-up period of one year postoperatively.

**Results:**

Taking into account that patients without mesh fixation had smaller defects and were treated with smaller meshes indicating non-equivalent groups, postoperative complications (general, intra- and postoperative complications, as well as complication-related reoperations), were similar among groups except that self-fixing meshes showed a lower general complication rate compared to fixed meshes (OR = 0.733 [0.579; 0.929]; *p* = 0.010). Mesh fixation had no relation to recurrence rate. Self-fixating meshes were associated with increased pain at rest rate (OR = 1.325 [1.156; 1.518]; *p* < 0.001), pain on exertion rate (OR = 1.255 [1.125; 1.400], *p* < 0.001) and chronic pain requiring treatment (OR = 1.271 [1.086; 1.488], *p* = 0.003) compared to fixed meshes. Self-fixating (OR = 1.675 [1.322; 2.120], *p* < 0.001) and fixed meshes (OR = 1.334 [1.069; 1.666], *p* = 0.011) were associated to increased pain on exertion rate compared to non-fixed meshes.

**Conclusion:**

It appears that mesh fixation can be omitted during sublay incisional hernia repair.

**Supplementary Information:**

The online version contains supplementary material available at 10.1007/s00423-025-03714-8.

## Introduction

Incisional hernias frequently occur after abdominal surgery [1, 2]. Depending on the surgical procedure, 4.6–22.4% of patients develop incisional hernias within three years [3, 4]. In most cases patients undergo surgical repair, as incisional hernias may be symptomatic, visually disturbing, and may lead to bowel incarceration [5, 6].

Among other (minimally invasive) methods, open sublay replair reflects the gold standard treatment for incisional hernias nowadays [7–10]. Many surgeons use sutures to keep the mesh in place [11, 12], while others use self-adhesive meshes [13] or forego fixation completely [7].

Ellis et al. [11] performed a noninferiority randomized clinical trial evaluating the effect of using no fixation on recurrence rates among patients undergoing open retromuscular ventral hernia repair. They found that it is safe to abandon transfascial mesh fixation in patients undergoing open retromuscular ventral hernia repair [11]. As this is a single center study with only 325 patients more data are needed [11].

The aim of this study is hence to assess the outcomes following open incisional hernia repair with sublay technique (including intra-, postoperative- and general complications, complication-related re-operations, recurrences, and chronic pain) in patients with and without mesh fixation.

## Materials and methods

The Herniamed quality assurance study [14, 15] is a multicentre, online, hernia registry. It was founded in the year 2009 with the goal to report on hernia surgery and respective outcome research. 836 participating hospitals and surgeons in private practice (Herniamed Study Group) in Germany, Switzerland and Austria, who have collected patient data and outcomes following hernia surgery, are included. All patients signed informed consent and were informed about the fact that the respective hospital or practice should be informed about potential problems occurring postoperatively as well as requiring clinical control when necessary. After 1-, 5- and 10 years patients and their general practitioners are sent a questionnaire by the treating surgeon or hospital, enquiring once again about any postoperative complications. In addition, in the questionnaire patients and their general practitioners are asked about any pain at rest, pain on exertion or chronic pain requiring treatment. It is also asked about any suspicious protrusion.

The following retrospective analysis aims to compare and assess the data collected for incisional hernias divided by different fixation techniques (no fixation, fixation, self-fixation) for intraoperative-, general- and postoperative complications, complication-related re-operations, recurrence and chronic pain.

The data analyses were performed with the software SAS 9.4 (SAS Institute Inc., Cary, NC, USA). They were calculated to a full significance level of 5%, meaning they were not corrected with multiple tests.

**Fig. 1:**
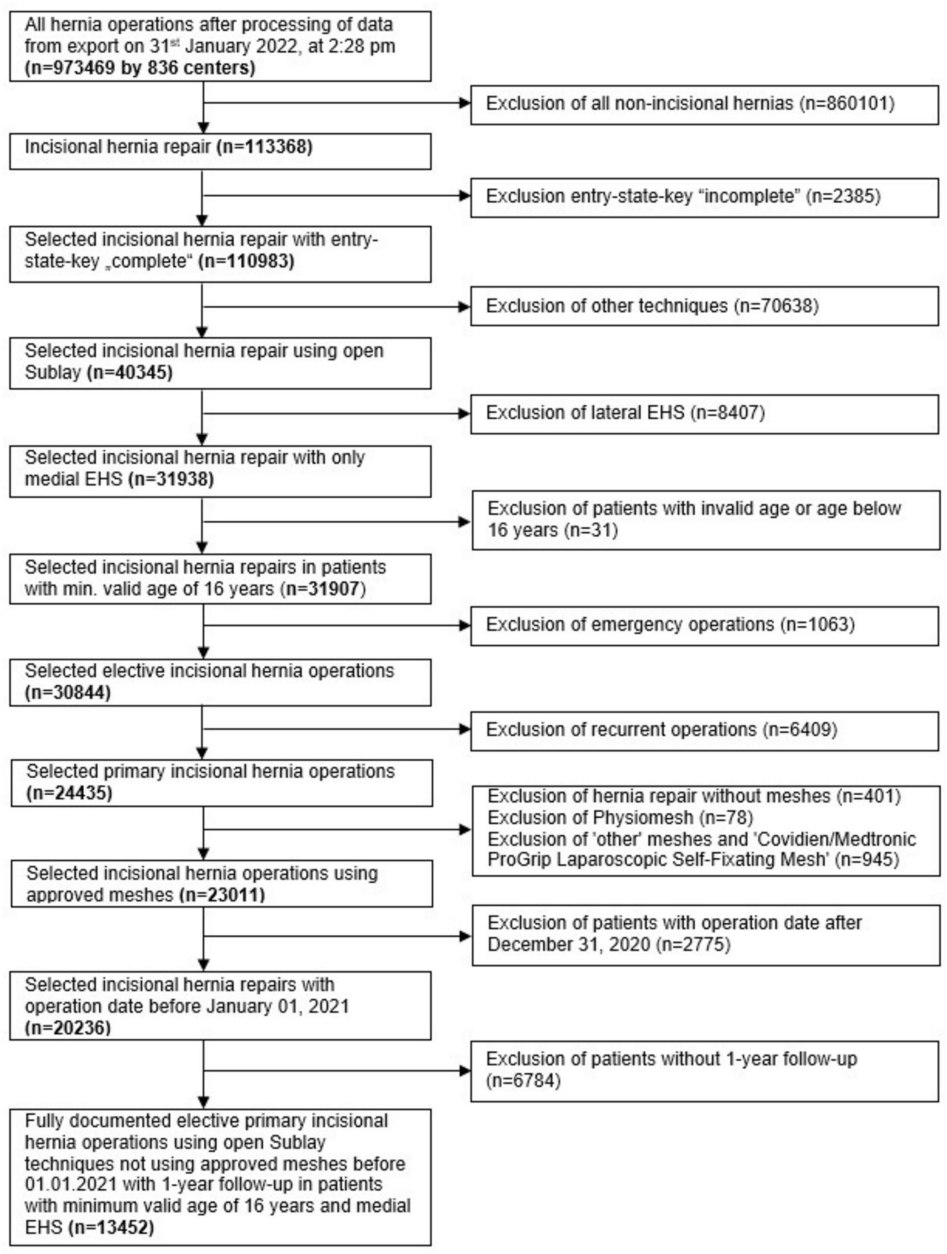
Flow chart for inclusion criteria

Amongst the 973’469 patients included in the database, the following items were used as inclusion criteria: surgery of an incisional hernia, open sublay technique, medial EHS, complete documentation of the patient and respective surgery, patient age ≥ 16 years, no emergency surgery, usage of an approved mesh, date of surgery until December 2020, available 1-year-follow-up data with complete documentation (Fig. 1).

Intraoperative complications included bleeding and organ injuries (i.e. injuries to vessels, bowel, bladder, stomach, spleen, liver etc.).

General complications included fever, urinary tract infections, diarrhea, gastritis, thrombosis, pulmonary embolism, pneumonia, chronic obstructive pulmonary disease (COPD), cardiac insufficiency, coronary heart disease, renal insufficiency, hypertensive crisis, and deceased patients.

Postoperative complications included bleeding, seroma formation, prolonged ileus, small bowel obstruction, bowel injury, anastomotic insufficiency, wound healing disorder and infection.

Unadjusted analysis was performed to analyse the relation of specific parameters to mesh fixation. Chi-square test and ANOVA was used for categorical and continuous outcome variables, respectively. Analyses of non-normal distributed data (duration of mesh size) was conducted on log-transformed values.

In addition to mesh fixation [no fixation / fixation / self-fixation], other potential influencing parameters were assessed in multivariable logistic regression models:

1. Age in years.
2. BMI in kg/m².
3. Mesh size in cm².
4. Gender [male / female].
5. ASA [I / II / III - IV.
6. Defect size [W1 (< 4 cm) / W2 ( > = 4–10 cm) / W3 (> 10 cm)]
7. Preoperative pain [yes / no / unknown].
8. Drains [yes / no].
9. Presence of risk factors [yes / no].

As well as postoperative complications for the analysis of pain at follow-up.

Risk factors apply if at least one of the following risk factors are present:

1. Chronic obstructive pulmonary disease (COPD).
2. Diabetes mellitus.
3. Aortic aneurysm.
4. Immunosuppression.
5. Corticosteroids.
6. Smoking.
7. Coagulopathy.
8. Platelet aggregation inhibitors (discontinued less than 7 days before surgery).
9. Coumarin derivates (Quick/INR not in normal range).

## Results

### Unadjusted analysis

As seen in the patient inclusion flowchart (Fig. 1), 13’452 patients with primary elective incisional hernia repair in open sublay technique were available for retrospective analysis of the prospectively collected data in the Herniamed-Registry. 9’986 patients (74.23%) were in the fixation group, 2’725 patients (20.26%) were in the self-fixation group, and 741 patients (5.51%) were in the no fixation group.

Mean body mass index (BMI) and age did not significantly differ between groups. In the non-fixation group mesh size was smaller when compared to the two other groups (*p* < 0.001) (Table 1).

**Table 1:** Compilation of simple ranges and results of unadjusted tests on the association of comparison groups with age, BMI, and mesh Size

		Mesh fixation			*p*
		No fixation	Fixation	Self Fixation	
Age [years]	N / Mean ± SD	741 / 62.7 ± 13.4	9986 / 63.5 ± 13.0	2725 / 63.4 ± 12.8	0.331
BMI [kg/m²]	N / Mean ± SD	739 / 29.4 ± 5.8	9950 / 29.2 ± 5.8	2719 / 29.1 ± 5.7	0.426
Mesh size [cm²]*	N / Mean [Range of dispersion]	741 / 238.6 [236.1; 241.0]	9983 / 277.2 [274.8; 279.6]	2725 / 272.9 [271.1; 274.7]	< 0.001

* Logarithmic transformation: illustration of the back-transformed mean values and ranges (mean value ± SD)

In addition, patients without mesh fixation had smaller defects (*p* < 0.001), more often suffered from preoperative pain (*p* < 0.001) and were less likely treated with drain insertion (*p* < 0.001). Other factors assessed (including gender, ASA score, as well as different risk factors) did not relate to the mesh fixation technique (Table 2).

**Table 2:** Results for the unadjusted tests between fixation group and the categorical variables

		Mesh fixation						*p*
		No fixation		Fixation		Self Fixation		
		*n*	%	*n*	%	*n*	%	
Gender	Male	382	51.6	5215	52.2	1442	52.9	0.740
	Female	359	48.4	4771	47.8	1283	47.1	
ASA	I	63	8.5	874	8.8	264	9.7	0.388
	II	438	59.1	5670	56.8	1545	56.7	
	III/IV	240	32.4	3442	34.5	916	33.6	
Defect size	I (< 4 cm)	257	34.7	2372	23.8	526	19.3	< 0.001
	II (4–10 cm)	376	50.7	5421	54.3	1660	60.9	
	III (> 10 cm)	108	14.6	2193	22.0	539	19.8	
Preoperative pain	no	242	32.7	3634	36.4	847	31.1	< 0.001
	yes	449	60.6	5576	55.8	1561	57.3	
	unknown	50	6.7	776	7.8	317	11.6	
Drainage	yes	529	71.4	8521	85.3	2417	88.7	< 0.001
	no	212	28.6	1465	14.7	308	11.3	
Risk factors - total	yes	305	41.2	4306	43.1	1176	43.2	0.575
	no	436	58.8	5680	56.9	1549	56.8	
- COPD	yes	67	9.0	1053	10.5	288	10.6	0.427
	no	674	91.0	8933	89.5	2437	89.4	
- Diabetes	yes	91	12.3	1319	13.2	335	12.3	0.383
	no	650	87.7	8667	86.8	2390	87.7	
- Aortic aneurysm	yes	12	1.6	227	2.3	68	2.5	0.364
	no	729	98.4	9759	97.7	2657	97.5	
- Immunosuppression	yes	15	2.0	193	1.9	55	2.0	0.951
	no	726	98.0	9793	98.1	2670	98.0	
- Corticoids	yes	14	1.9	164	1.6	36	1.3	0.395
	no	727	98.1	9822	98.4	2689	98.7	
- Smoking	yes	107	14.4	1321	13.2	356	13.1	0.608
	no	634	85.6	8665	86.8	2369	86.9	
- Coagulopathy	yes	16	2.2	237	2.4	65	2.4	0.931
	no	725	97.8	9749	97.6	2660	97.6	
- Antithrombotic medication	yes	94	12.7	1370	13.7	379	13.9	0.688
	no	647	87.3	8616	86.3	2346	86.1	
- Anticoagulant medication	yes	26	3.5	340	3.4	104	3.8	0.584
	no	715	96.5	9646	96.6	2621	96.2	

Furthermore, the unadjusted analysis showed most general complications occurring in the mesh fixation group (4.6% vs. 3.2% (no fixation) vs. 3.2% (self-fixation), *p* = 0.003), the lowest incidence of pain on exertion at 1-year follow-up in the no fixation group (13.6% vs. 17.1% (fixation) vs. 20.6% (self-fixation), *p* < 0.001), the highest incidence of pain at rest at 1-year follow-up in the self-fixation group (12.1% vs. 10.4% (no fixation) vs. 9.4% (fixation), *p* < 0.001), as well as the highest incidence of pain requiring treatment at 1-year follow-up in the self-fixation group (8.6% vs. 7.0% (no fixation) vs. 6.9% (fixation), *p* 0.009) (Table 3).

**Table 3:** Results for the unadjusted tests between fixation group and outcome variables

		Mesh fixation						*p*
		No fixation		Fixation		Self Fixation		
		*n*	%	*n*	%	*n*	%	
Intraoperative complications	yes	10	1.3	145	1.5	42	1.5	0.909
	no	731	98.7	9841	98.5	2683	98.5	
General complications	yes	24	3.2	460	4.6	88	3.2	0.003
	no	717	96.8	9526	95.4	2637	96.8	
Postoperative complications	yes	69	9.3	1070	10.7	289	10.6	0.489
	no	672	90.7	8916	89.3	2436	89.4	
Complication-related reoperations	yes	22	3.0	486	4.9	135	5.0	0.058
	no	719	97.0	9500	95.1	2590	95.0	
Recurrence on 1-year follow-up	yes	21	2.8	329	3.3	108	4.0	0.158
	no	720	97.2	9657	96.7	2617	96.0	
Pain on exertion on 1-year follow-up	yes	101	13.6	1708	17.1	560	20.6	< 0.001
	no	640	86.4	8278	82.9	2165	79.4	
Pain at rest on 1-year follow-up	yes	77	10.4	940	9.4	329	12.1	< 0.001
	no	664	89.6	9046	90.6	2396	87.9	
Pain requiring treatment on 1-year follow-up	yes	52	7.0	689	6.9	235	8.6	0.009
	no	689	93.0	9297	93.1	2490	91.4	

### Multivariable analyses

#### Intraoperative complications

Intraoperative complications occurred more frequently when larger meshes were used (OR = 1.735 [1.343; 2.241]; *p* < 0.001). The intraoperative complication rate was not significantly affected by other variables assessed including mesh fixation technique (Suppl. Table 1).

#### Postoperative complications

The use of larger meshes (OR = 1.70 [1.550; 1.877]; *p* < 0.001), the presence of at least one risk factor (OR = 1.532 [1.361; 1.723]; *p* < 0.001), a higher BMI (5-points-OR = 1.138 [1.085; 1.194]; *p* < 0.001), a larger defect (OR = 1.23 [1.072; 1.400]; *p* = 0.011), and preoperative pain (OR = 1.153 [1.020; 1.303]; *p* = 0.035) were significantly associated with an increased risk of postoperative complications. The postoperative complication rate was not significantly affected by the mesh fixation technique (Suppl. Table 2).

#### Complication-related reoperations

A larger mesh (OR = 1.865 [1.619; 2.148]; *p* < 0.001), the presence of at least one risk factor (OR = 1.608 [1.356; 1.907]; *p* < 0.001), a higher BMI (5-points-OR = 1.115 [1.042; 1.192]; *p* = 0.002), and an ASA score of III/IV vs. II (OR = 1.260 [1.053; 1.508]; *p* = 0.012) were significantly associated with an increased risk of complication-related reoperations. The mesh fixation technique only showed a tendency to be associated with complication-related reoperations (*p* = 0.096) with self-fixing meshes leading to approx. 57 / 1000 complication-related reoperations versus approx. 35 / 1000 without mesh fixation (OR = 1.665 [1.048; 2.646]; *p* = 0.031) (Suppl. Table 3).

#### General complications

The use of larger meshes (OR = 1.710 [1.474; 1.983]; *p* < 0.001), higher age (10-years-OR = 1.253 [1.157; 1.357]; *p* < 0.001), the presence of at least one risk factor (OR = 1.409 [1.178; 1.685]; *p* < 0.001), an ASA score of III/IV vs. II (OR = 1.361 [1.129; 1.641]; *p* < 0.001), presence of preoperative pain vs. no pain (OR = 1.322 [1.095; 1.596]; *p* = 0.004), presence of preoperative pain vs. unknown (OR = 1.454 [1.027; 2.059]; *p* = 0.035) and mesh fixation technique (*p* = 0.022) (with self-fixing meshes having a lower general complication rate compared to fixed meshes (OR = 0.733 [0.579; 0.929]; *p* = 0.010) were significantly associated with an increased risk of general complications (Suppl. Table 4). The latter corresponds to approx. 37 general complications in 1000 operations with self-fixing meshes compared to approx. 50 / 1000 operations with fixation (prevalence of ∼ 4.3%).

#### Recurrence

Recurrence risk at 1-year follow-up was higher in patients with higher BMI (5-points-OR = 1.143 [1.056; 1.238]; *p* = 0.001), when smaller meshes were used (OR = 0.807 [0.701; 0.929]; *p* = 0.003), in females (OR = 0.756 [0.623; 0.917]; *p* = 0.004) and in patients with larger defects (*p* = 0.036) (with defects > 10 cm vs. < 4 cm (OR = 1.554 [1.102; 2.191]; *p* = 0.012) and defects 4–10 cm vs. < 4 cm (OR = 1.365 [1.037; 1.797]; *p* = 0.027) showing a higher recurrence rate). Mesh fixation technique was had no significant relation to risk of recurrence (Suppl. Table 5).

#### Pain at rest

The following factors were significantly associated with increased pain at rest after surgery: lower patients’ age (10-years-OR = 0.831 [0.793; 0.871]; *p* < 0.001), occurrence of postoperative complications (OR = 1.822 [1.554; 2.135]; *p* < 0.001), female gender (OR = 1.550 [1.379; 1.742]; *p* < 0.001), the presence of preoperative pain vs. no pain (OR = 1.635 [1.431; 1.869]; *p* < 0.001), unknown vs. no preoperative pain (OR = 1.397 [1.115; 1.751]; *p* = 0.004), use of larger meshes (OR = 1.288 [1.175; 1.412]; *p* < 0.001), lower BMI (5-points-OR = 0.906 [0.861; 0.952]; *p* < 0.001), and mesh fixation technique (*p* < 0.001) (with self-fixating meshes being associated with increased pain at rest compared to fixed meshes (OR = 1.325 [1.156; 1.518]; *p* < 0.001) (Suppl. Table 6). The latter corresponds to approx. 112 patients with pain at rest at 1-year follow-up in 1000 operations with self-fixing meshes compared to approx. 87 / 1000 operations with fixation (prevalence of ∼ 9.96%).

#### Pain on exertion

The following factors were significantly associated with increased pain on exertion on follow-up one year after surgery: lower patients’ age (10-years-OR = 0.763 [0.735; 0.792]; *p* < 0.001), female gender (OR = 1.625 [1.482; 1.783]; *p* < 0.001), the presence of preoperative pain vs. no pain (OR = 1.538 [1.387; 1.705]; *p* < 0.001), unknown vs. no preoperative pain (OR = 1.298 [1.086; 1.552]; *p* = 0.004), the presence of preoperative pain vs. unknown (OR = 1.184 [1.002; 1.400]; *p* = 0.047), occurrence of postoperative complications (OR = 1.513 [1.321; 1.734]; *p* < 0.001), use of larger meshes (OR = 1.242 [1.156; 1.333]; *p* < 0.001), lower BMI (5-points-OR = 0.930 [0.894; 0.968]; *p* < 0.001), and the presence of at least one risk factor (OR = 1.151 [1.045; 1.267]; *p* = 0.004). Likewise, mesh fixation technique was associated with increased pain on exertion (*p* < 0.001) with self-fixating meshes being associated with increased pain on exertion compared to non-fixed meshes (OR = 1.675 [1.322; 2.120]; *p* < 0.001; approx. 230/1000 vs. 151/1000) and to fixed meshes (OR = 1.255 [1.125; 1.400]; *p* < 0.001; approx. 195/1000 vs. 162/1000), respectively, and fixed meshes being associated with increased pain on exertion compared to non-fixed meshes (OR = 1.334 [1.069; 1.666], *p* = 0.011; approx. 169/1000 vs. 149/1000) (Suppl. Table 7).

#### Chronic pain requiring treatment

The following factors were significantly associated with chronic pain requiring treatment: lower patients’ age (10-years-OR = 0.782 [0.741; 0.825]; *p* < 0.001), presence of preoperative pain vs. no pain (OR = 1.962 [1.670; 2.304]; *p* < 0.001), unknown vs. no preoperative pain (OR = 1.440 [1.095; 1.896]; *p* = 0.009), the presence of preoperative pain vs. unknown (OR = 1.362 [1.061; 1.748]; *p* = 0.015), female gender (OR = 1.606 [1.403; 1.839]; *p* < 0.001), occurrence of postoperative complications (OR = 1.731 [1.442; 2.078]; *p* < 0.001), use of larger meshes (OR = 1.251 [1.126; 1.390]; *p* < 0.001), the presence of at least one risk factor (OR = 1.269 [1.103; 1.460]; *p* < 0.001), lower BMI (5-points-OR = 0.926 [0.875; 0.979]; *p* = 0.007), and mesh fixation technique (*p* = 0.011) (with self-fixating meshes being associated with increased chronic pain requiring treatment compared to fixed meshes (OR = 1.271 [1.086; 1.488]; *p* = 0.003). This corresponds to approx. 81 / 1000 cases of pain requiring treatment at 1-year follow-up with self-fixing meshes compared to approx. 65 /1000 with fixed meshes (Suppl. Table 8).

#### Standardized differences for patients with and without follow-up

**Fig. 2:**
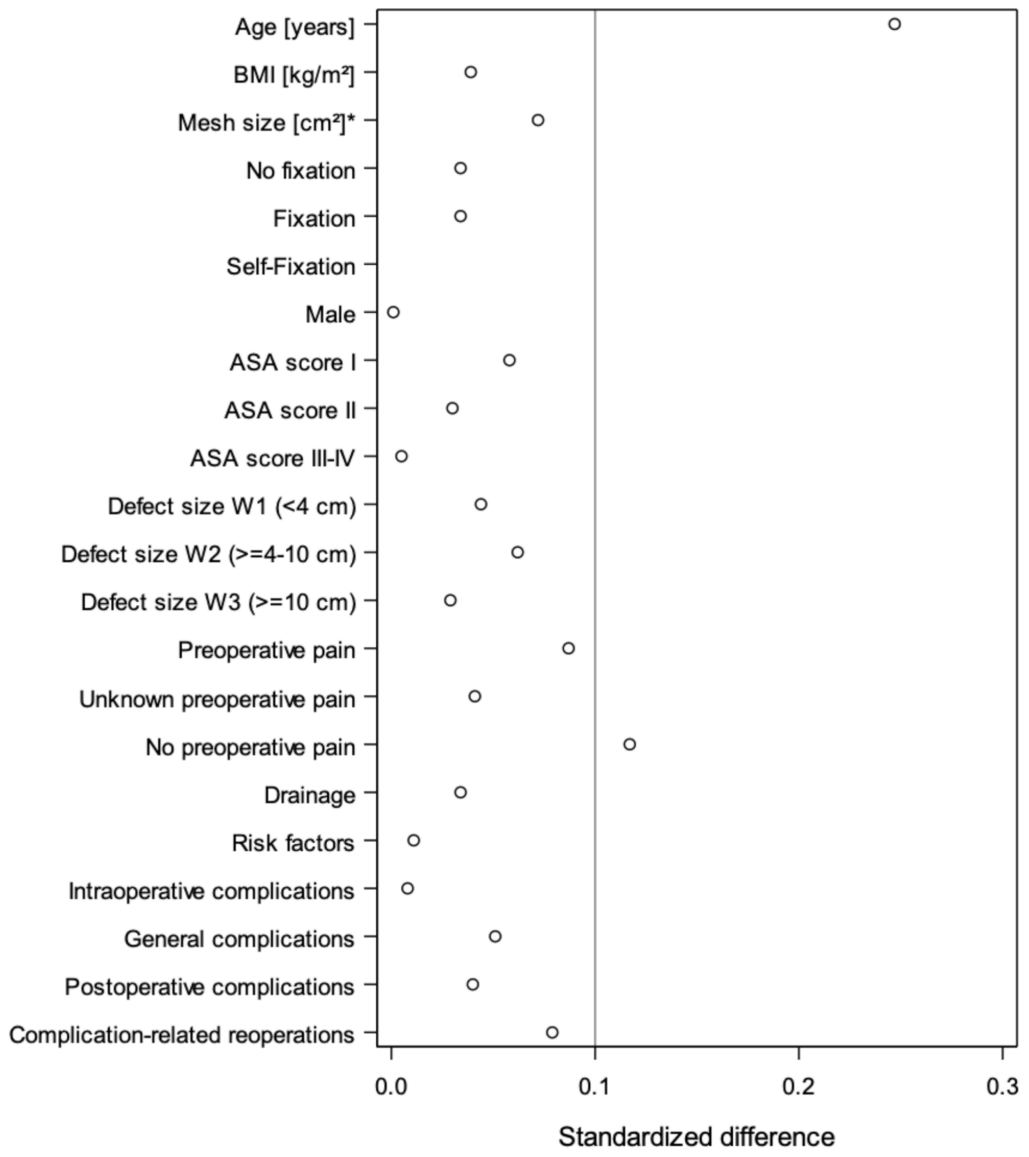
Scatter plot of standardized differences between patients with and without follow-up

Figure 2 demonstrates the standardized differences for patients with (*n* = 13.452) and without (*n* = 6.784) follow-up-information. Standardized differences above the value of 10% were only found for age and no preoperative pain. For all other variables including the complication rates standardized differences below 0.1% were found. A selection of patients as bias can therefore be excluded.

## Discussion

In this retrospective analysis of prospective data, the Herniamed online register was used to investigate whether it is possible to completely avoid mesh fixation when treating incisional hernias using the open sublay method. Three groups of patients were compared: those in whom the mesh was fixed (*n* = 9’986), those in whom a self-fixing mesh was used (*n* = 2’725), and those in whom no mesh fixation was performed (*n* = 741).

Regarding postoperative complications (general, intra- and postoperative complications, as well as complication-related reoperations), no significant differences were found for the different mesh fixation variants except that self-fixing meshes showed a lower general complication rate compared to fixed meshes. In this respect, the no-fixation technique is not significantly different to mesh fixation and use of self-fixing meshes. In the study of the Cleveland Clinic [11] at 30 days, there was a significant difference in the surgical site occurrence (SSO) rates (5.2% in the fixation group vs. 15.9% in the non fixation group, *p* = 0.007).

Our own study does not show any significant difference in the rates of postoperative complications and complication-related reoperations up to day 30 between the non-fixation, self-fixation and fixation techniques. Differences in the perioperative outcome may be explained by the different study design (i.e. data from an online registry versus a randomized controlled trial) and by the fact that the collective of the study by Ellis [11] showed a higher recurrence rate when compared to the collective of patients from the Herniamed Registry.

The occurrence of recurrences in the 1-year follow-up was also not significantly associated with mesh fixation. Therefore, all three fixation variants can be interpreted as comparable with regard to recurrence.

Regarding postoperative pain, self-fixating meshes were significantly associated with increased pain at rest, pain on exertion and chronic pain requiring treatment compared to fixed meshes. Furthermore, self-fixating and fixed meshes were significantly associated with increased pain on exertion compared to non-fixed meshes. Hence, regarding postoperative pain, self-fixating meshes should be avoided. Furthermore, not to fix the mesh is considered superior to fixed or self-fixating meshes, respectively.

In general, the present study is limited by the fact, that it is based on an online registry. A further limitation is the rather small percentage of patients treated without mesh fixation (*n* = 741, 5.5%) possibly indicating that this is a non-comparable group with a less pronounced defect. Our observations may be confounded by further (un-)observed confounders.

In a systematic review using self-gripping mesh most of the reviewed articles demonstrate that this is a safe and effective method for repairing incisional hernias in sublay technique [13].

## Conclusion

It appears that mesh fixation can be omitted during sublay incisional hernia repair. To check whether this finding is actually true or whether it is attributable to an unequal group distribution (i.e. meshes were less likely to be fixed in patients with minor defects), randomized prospective trials comparing mesh fixation methods for incisional hernia repair are needed.

## Electronic supplementary material

Below is the link to the electronic supplementary material.

Supplementary Material 1

## Data Availability

No datasets were generated or analysed during the current study.
