# Supplementary material for: Effect of mesh fixation in incisional hernia repair using the open sublay technique: results from the herniamed-registry
Source: Langenbecks Arch Surg. 2025 Apr 23;410(1):141. doi: 10.1007/s00423-025-03714-8 (PMC12018522; doi:10.1007/s00423-025-03714-8)
Supplement: Supplementary file 1 — Supplementary Material 1 [file 423_2025_3714_MOESM1_ESM.docx]

| **Variable** | **p-Value** | **Categories** | **Odds Ratio** | **LCL** | **UCL** | **p-Value (pair- wise)** |
| --- | --- | --- | --- | --- | --- | --- |
|  | | | | | | |
| Mesh size [cm²]* | <0.001 |  | 1.735 | 1.343 | 2.241 |  |
|  | | | | | | |
| BMI [5-points-OR] | 0.098 |  | 0.894 | 0.783 | 1.021 |  |
|  | | | | | | |
| Defect size | 0.137 | III (> 10 cm) vs I (< 4 cm) | 1.741 | 1.006 | 3.014 | 0.048 |
|  |  | II (4 - 10 cm) vs I (< 4 cm) | 1.540 | 0.945 | 2.510 | 0.083 |
|  |  | III (> 10 cm) vs II (4 - 10 cm) | 1.131 | 0.815 | 1.569 | 0.462 |
|  | | | | | | |
| Age [10-years-OR] | 0.163 |  | 1.094 | 0.964 | 1.240 |  |
|  | | | | | | |
| Gender | 0.316 | Female vs Male | 1.158 | 0.870 | 1.541 |  |
|  | | | | | | |
| Drainage | 0.371 | yes vs no | 1.283 | 0.743 | 2.216 |  |
|  | | | | | | |
| ASA | 0.585 | III/IV vs II | 1.136 | 0.825 | 1.563 | 0.435 |
|  |  | II vs I | 0.804 | 0.465 | 1.390 | 0.435 |
|  |  | III/IV vs I | 0.913 | 0.503 | 1.655 | 0.764 |
|  | | | | | | |
| Preoperative pain | 0.695 | yes vs no | 1.040 | 0.764 | 1.416 | 0.805 |
|  |  | unknown vs no | 1.245 | 0.751 | 2.064 | 0.396 |
|  |  | yes vs unknown | 0.835 | 0.515 | 1.355 | 0.465 |
|  | | | | | | |
| Mesh fixation | 0.825 | Self-Fixation vs Fixation | 1.112 | 0.783 | 1.579 | 0.552 |
|  |  | Fixation vs No fixation | 0.921 | 0.481 | 1.764 | 0.804 |
|  |  | Self-Fixation vs No fixation | 1.025 | 0.509 | 2.063 | 0.946 |
|  | | | | | | |
| Risk factors | 0.875 | yes vs no | 1.024 | 0.760 | 1.381 |  |

**Suppl. Table 1:** A Multivariable analysis results for intraoperative complications, including odds ratio estimates with corresponding 95% confidence intervals.

* Logarithmic transformation

| **Variable** | **p-Value** | **Categories** | **Odds Ratio** | **LCL** | **UCL** | **p-Value (pair- wise)** |
| --- | --- | --- | --- | --- | --- | --- |
|  | | | | | | |
| Mesh size [cm²]* | <0.001 |  | 1.706 | 1.550 | 1.877 |  |
|  | | | | | | |
| Risk factors | <0.001 | yes vs no | 1.532 | 1.361 | 1.723 |  |
|  | | | | | | |
| BMI [5-points-OR] | <0.001 |  | 1.138 | 1.085 | 1.194 |  |
|  | | | | | | |
| Defect size | 0.011 | III (> 10 cm) vs II (4 - 10 cm) | 1.225 | 1.072 | 1.400 | 0.003 |
|  |  | III (> 10 cm) vs I (< 4 cm) | 1.200 | 0.987 | 1.459 | 0.067 |
|  |  | II (4 - 10 cm) vs I (< 4 cm) | 0.980 | 0.829 | 1.158 | 0.811 |
|  | | | | | | |
| Preoperative pain | 0.035 | yes vs no | 1.153 | 1.020 | 1.303 | 0.023 |
|  |  | yes vs unknown | 1.201 | 0.969 | 1.488 | 0.094 |
|  |  | unknown vs no | 0.960 | 0.768 | 1.200 | 0.720 |
|  | | | | | | |
| Age [10-years-OR] | 0.064 |  | 1.048 | 0.997 | 1.101 |  |
|  | | | | | | |
| ASA | 0.101 | III/IV vs II | 1.144 | 1.009 | 1.297 | 0.036 |
|  |  | II vs I | 0.923 | 0.732 | 1.163 | 0.495 |
|  |  | III/IV vs I | 1.055 | 0.823 | 1.354 | 0.671 |
|  | | | | | | |
| Gender | 0.205 | Female vs Male | 0.929 | 0.828 | 1.041 |  |
|  | | | | | | |
| Drainage | 0.224 | yes vs no | 0.890 | 0.737 | 1.074 |  |
|  | | | | | | |
| Mesh fixation | 0.609 | Self-Fixation vs No fixation | 1.140 | 0.860 | 1.511 | 0.362 |
|  |  | Self-Fixation vs Fixation | 1.053 | 0.915 | 1.212 | 0.468 |
|  |  | Fixation vs No fixation | 1.082 | 0.834 | 1.404 | 0.553 |

**Suppl. Table 2:** Multivariable analysis results for postoperative complications, including odds ratio estimates with corresponding 95% confidence intervals.

* Logarithmic transformation

| **Variable** | **p-Value** | **Categories** | **Odds Ratio** | **LCL** | **UCL** | **p-Value (pair- wise)** |
| --- | --- | --- | --- | --- | --- | --- |
|  | | | | | | |
| Mesh size [cm²]* | <0.001 |  | 1.865 | 1.619 | 2.148 |  |
|  | | | | | | |
| Risk factors | <0.001 | yes vs no | 1.608 | 1.356 | 1.907 |  |
|  | | | | | | |
| BMI [5-points-OR] | 0.002 |  | 1.115 | 1.042 | 1.192 |  |
|  | | | | | | |
| ASA | 0.041 | III/IV vs II | 1.260 | 1.053 | 1.508 | 0.012 |
|  |  | III/IV vs I | 1.159 | 0.809 | 1.661 | 0.420 |
|  |  | II vs I | 0.920 | 0.657 | 1.288 | 0.627 |
|  | | | | | | |
| Preoperative pain | 0.057 | yes vs no | 1.239 | 1.036 | 1.480 | 0.019 |
|  |  | yes vs unknown | 1.150 | 0.850 | 1.556 | 0.364 |
|  |  | unknown vs no | 1.077 | 0.783 | 1.480 | 0.648 |
|  | | | | | | |
| Mesh fixation | 0.096 | Self-Fixation vs No fixation | 1.665 | 1.048 | 2.646 | 0.031 |
|  |  | Fixation vs No fixation | 1.522 | 0.982 | 2.358 | 0.060 |
|  |  | Self-Fixation vs Fixation | 1.094 | 0.896 | 1.336 | 0.377 |
|  | | | | | | |
| Defect size | 0.137 | III (> 10 cm) vs II (4 - 10 cm) | 1.185 | 0.980 | 1.432 | 0.080 |
|  |  | II (4 - 10 cm) vs I (< 4 cm) | 0.857 | 0.675 | 1.088 | 0.205 |
|  |  | III (> 10 cm) vs I (< 4 cm) | 1.015 | 0.771 | 1.337 | 0.915 |
|  | | | | | | |
| Drainage | 0.429 | yes vs no | 1.128 | 0.837 | 1.518 |  |
|  | | | | | | |
| Age [10-years-OR] | 0.587 |  | 0.981 | 0.914 | 1.052 |  |
|  | | | | | | |
| Gender | 0.621 | Female vs Male | 0.959 | 0.814 | 1.131 |  |

**Suppl. Table 3:** Multivariable analysis results for complication-related reoperations, including odds ratio estimates with corresponding 95% confidence intervals.

* Logarithmic transformation

| **Variable** | **p-Value** | **Categories** | **Odds Ratio** | **LCL** | **UCL** | **p-Value (pair- wise)** |
| --- | --- | --- | --- | --- | --- | --- |
|  | | | | | | |
| Mesh size [cm²]* | <0.001 |  | 1.710 | 1.474 | 1.983 |  |
|  | | | | | | |
| Age [10-years-OR] | <0.001 |  | 1.253 | 1.157 | 1.357 |  |
|  | | | | | | |
| Risk factors | <0.001 | yes vs no | 1.409 | 1.178 | 1.685 |  |
|  | | | | | | |
| ASA | 0.004 | III/IV vs II | 1.361 | 1.129 | 1.641 | 0.001 |
|  |  | III/IV vs I | 1.473 | 0.953 | 2.275 | 0.081 |
|  |  | II vs I | 1.082 | 0.713 | 1.643 | 0.712 |
|  | | | | | | |
| Preoperative pain | 0.004 | yes vs no | 1.322 | 1.095 | 1.596 | 0.004 |
|  |  | yes vs unknown | 1.454 | 1.027 | 2.059 | 0.035 |
|  |  | unknown vs no | 0.909 | 0.632 | 1.308 | 0.608 |
|  | | | | | | |
| Mesh fixation | 0.022 | Self-Fixation vs Fixation | 0.733 | 0.579 | 0.929 | 0.010 |
|  |  | Fixation vs No fixation | 1.307 | 0.857 | 1.994 | 0.214 |
|  |  | Self-Fixation vs No fixation | 0.958 | 0.602 | 1.525 | 0.857 |
|  | | | | | | |
| Gender | 0.151 | Female vs Male | 1.135 | 0.955 | 1.349 |  |
|  | | | | | | |
| Defect size | 0.157 | III (> 10 cm) vs II (4 - 10 cm) | 1.210 | 0.992 | 1.477 | 0.060 |
|  |  | III (> 10 cm) vs I (< 4 cm) | 1.224 | 0.906 | 1.655 | 0.189 |
|  |  | II (4 - 10 cm) vs I (< 4 cm) | 1.012 | 0.777 | 1.316 | 0.932 |
|  | | | | | | |
| Drainage | 0.479 | yes vs no | 1.120 | 0.819 | 1.530 |  |
|  | | | | | | |
| BMI [5-points-OR] | 0.633 |  | 1.019 | 0.944 | 1.099 |  |

**Suppl. Table 4:** Multivariable analysis results for general complications, including odds ratio estimates with corresponding 95% confidence intervals.

* Logarithmic transformation

| **Variable** | **p-Value** | **Categories** | **Odds Ratio** | **LCL** | **UCL** | **p-Value (pair- wise)** |
| --- | --- | --- | --- | --- | --- | --- |
|  | | | | | | |
| BMI [5-points-OR] | 0.001 |  | 1.143 | 1.056 | 1.238 |  |
|  | | | | | | |
| Mesh size [cm²]* | 0.003 |  | 0.807 | 0.701 | 0.929 |  |
|  | | | | | | |
| Gender | 0.004 | Female vs Male | 0.756 | 0.623 | 0.917 |  |
|  | | | | | | |
| Defect size | 0.036 | III (> 10 cm) vs I (< 4 cm) | 1.554 | 1.102 | 2.191 | 0.012 |
|  |  | II (4 - 10 cm) vs I (< 4 cm) | 1.365 | 1.037 | 1.797 | 0.027 |
|  |  | III (> 10 cm) vs II (4 - 10 cm) | 1.138 | 0.895 | 1.448 | 0.291 |
|  | | | | | | |
| Mesh fixation | 0.276 | Self-Fixation vs Fixation | 1.178 | 0.941 | 1.473 | 0.152 |
|  |  | Self-Fixation vs No fixation | 1.338 | 0.829 | 2.159 | 0.234 |
|  |  | Fixation vs No fixation | 1.136 | 0.724 | 1.782 | 0.579 |
|  | | | | | | |
| ASA | 0.278 | III/IV vs II | 1.188 | 0.959 | 1.471 | 0.116 |
|  |  | III/IV vs I | 1.210 | 0.812 | 1.804 | 0.349 |
|  |  | II vs I | 1.019 | 0.709 | 1.466 | 0.919 |
|  | | | | | | |
| Drainage | 0.284 | yes vs no | 1.182 | 0.871 | 1.604 |  |
|  | | | | | | |
| Risk factors | 0.643 | yes vs no | 0.954 | 0.782 | 1.164 |  |
|  | | | | | | |
| Preoperative pain | 0.882 | yes vs no | 1.053 | 0.859 | 1.292 | 0.620 |
|  |  | yes vs unknown | 1.032 | 0.732 | 1.455 | 0.858 |
|  |  | unknown vs no | 1.021 | 0.713 | 1.461 | 0.912 |
|  | | | | | | |
| Age [10-years-OR] | 0.932 |  | 1.004 | 0.926 | 1.088 |  |

**Suppl. Table 5:** Multivariable analysis for recurrence, including odds ratio estimated with corresponding 95% confidence intervals.

* Logarithmic transformation

| **Variable** | **p-Value** | **Categories** | **Odds Ratio** | **LCL** | **UCL** | **p-Value (pair- wise)** |
| --- | --- | --- | --- | --- | --- | --- |
|  | | | | | | |
| Age [10-years-OR] | <0.001 |  | 0.831 | 0.793 | 0.871 |  |
|  | | | | | | |
| Postoperative complications | <0.001 | yes vs no | 1.822 | 1.554 | 2.135 |  |
|  | | | | | | |
| Gender | <0.001 | Female vs Male | 1.550 | 1.379 | 1.742 |  |
|  | | | | | | |
| Preoperative pain | <0.001 | yes vs no | 1.635 | 1.431 | 1.869 | <0.001 |
|  |  | unknown vs no | 1.397 | 1.115 | 1.751 | 0.004 |
|  |  | yes vs unknown | 1.170 | 0.950 | 1.442 | 0.139 |
|  | | | | | | |
| Mesh size [cm²]* | <0.001 |  | 1.288 | 1.175 | 1.412 |  |
|  | | | | | | |
| BMI [5-points-OR] | <0.001 |  | 0.906 | 0.861 | 0.952 |  |
|  | | | | | | |
| Mesh fixation | <0.001 | Self-Fixation vs Fixation | 1.325 | 1.156 | 1.518 | <0.001 |
|  |  | Self-Fixation vs No fixation | 1.178 | 0.900 | 1.543 | 0.232 |
|  |  | Fixation vs No fixation | 0.889 | 0.692 | 1.143 | 0.360 |
|  | | | | | | |
| Risk factors | 0.108 | yes vs no | 1.105 | 0.978 | 1.248 |  |
|  | | | | | | |
| ASA | 0.682 | III/IV vs I | 1.112 | 0.877 | 1.410 | 0.381 |
|  |  | II vs I | 1.081 | 0.876 | 1.334 | 0.468 |
|  |  | III/IV vs II | 1.029 | 0.899 | 1.176 | 0.682 |
|  | | | | | | |
| Defect size | 0.826 | III (> 10 cm) vs II (4 - 10 cm) | 0.958 | 0.827 | 1.110 | 0.569 |
|  |  | II (4 - 10 cm) vs I (< 4 cm) | 1.027 | 0.874 | 1.207 | 0.746 |
|  |  | III (> 10 cm) vs I (< 4 cm) | 0.984 | 0.804 | 1.204 | 0.876 |
|  | | | | | | |
| Drainage | 0.977 | yes vs no | 1.003 | 0.834 | 1.206 |  |

**Suppl. Table 6:** Multivariable analysis for pain at rest, including odds ratio estimated with corresponding 95% confidence intervals.

* Logarithmic transformation

| **Variable** | **p-Value** | **Categories** | **Odds Ratio** | **LCL** | **UCL** | **p-Value (pair- wise)** |
| --- | --- | --- | --- | --- | --- | --- |
|  | | | | | | |
| Age [10-years-OR] | <0.001 |  | 0.763 | 0.735 | 0.792 |  |
|  | | | | | | |
| Gender | <0.001 | Female vs Male | 1.625 | 1.482 | 1.783 |  |
|  | | | | | | |
| Preoperative pain | <0.001 | yes vs no | 1.538 | 1.387 | 1.705 | <0.001 |
|  |  | unknown vs no | 1.298 | 1.086 | 1.552 | 0.004 |
|  |  | yes vs unknown | 1.184 | 1.002 | 1.400 | 0.047 |
|  | | | | | | |
| Postoperative complications | <0.001 | yes vs no | 1.513 | 1.321 | 1.734 |  |
|  | | | | | | |
| Mesh size [cm²]* | <0.001 |  | 1.242 | 1.156 | 1.333 |  |
|  | | | | | | |
| Mesh fixation | <0.001 | Self-Fixation vs No fixation | 1.675 | 1.322 | 2.120 | <0.001 |
|  |  | Self-Fixation vs Fixation | 1.255 | 1.125 | 1.400 | <0.001 |
|  |  | Fixation vs No fixation | 1.334 | 1.069 | 1.666 | 0.011 |
|  | | | | | | |
| BMI [5-points-OR] | <0.001 |  | 0.930 | 0.894 | 0.968 |  |
|  | | | | | | |
| Risk factors | 0.004 | yes vs no | 1.151 | 1.045 | 1.267 |  |
|  | | | | | | |
| Drainage | 0.320 | yes vs no | 1.077 | 0.930 | 1.247 |  |
|  | | | | | | |
| Defect size | 0.403 | III (> 10 cm) vs I (< 4 cm) | 0.897 | 0.764 | 1.052 | 0.181 |
|  |  | III (> 10 cm) vs II (4 - 10 cm) | 0.943 | 0.837 | 1.062 | 0.335 |
|  |  | II (4 - 10 cm) vs I (< 4 cm) | 0.951 | 0.838 | 1.078 | 0.432 |
|  | | | | | | |
| ASA | 0.610 | III/IV vs II | 0.959 | 0.861 | 1.068 | 0.445 |
|  |  | II vs I | 1.061 | 0.902 | 1.247 | 0.476 |
|  |  | III/IV vs I | 1.017 | 0.845 | 1.224 | 0.858 |

**Suppl. Table 7:** Multivariable analysis for pain on exertion, including odds ratio estimated with corresponding 95% confidence intervals.

* Logarithmic transformation

| **Variable** | **p-Value** | **Categories** | **Odds Ratio** | **LCL** | **UCL** | **p-Value (pair- wise)** |
| --- | --- | --- | --- | --- | --- | --- |
|  | | | | | | |
| Age [10-years-OR] | <0.001 |  | 0.782 | 0.741 | 0.825 |  |
|  | | | | | | |
| Preoperative pain | <0.001 | yes vs no | 1.962 | 1.670 | 2.304 | <0.001 |
|  |  | unknown vs no | 1.440 | 1.095 | 1.896 | 0.009 |
|  |  | yes vs unknown | 1.362 | 1.061 | 1.748 | 0.015 |
|  | | | | | | |
| Gender | <0.001 | Female vs Male | 1.606 | 1.403 | 1.839 |  |
|  | | | | | | |
| Postoperative complications | <0.001 | yes vs no | 1.731 | 1.442 | 2.078 |  |
|  | | | | | | |
| Mesh size [cm²]* | <0.001 |  | 1.251 | 1.126 | 1.390 |  |
|  | | | | | | |
| Risk factors | <0.001 | yes vs no | 1.269 | 1.103 | 1.460 |  |
|  | | | | | | |
| BMI [5-points-OR] | 0.007 |  | 0.926 | 0.875 | 0.979 |  |
|  | | | | | | |
| Mesh fixation | 0.011 | Self-Fixation vs Fixation | 1.271 | 1.086 | 1.488 | 0.003 |
|  |  | Self-Fixation vs No fixation | 1.243 | 0.904 | 1.708 | 0.181 |
|  |  | Fixation vs No fixation | 0.977 | 0.726 | 1.316 | 0.880 |
|  | | | | | | |
| ASA | 0.078 | III/IV vs I | 1.339 | 1.014 | 1.769 | 0.040 |
|  |  | III/IV vs II | 1.150 | 0.986 | 1.341 | 0.075 |
|  |  | II vs I | 1.165 | 0.909 | 1.494 | 0.229 |
|  | | | | | | |
| Drainage | 0.374 | yes vs no | 1.104 | 0.888 | 1.373 |  |
|  | | | | | | |
| Defect size | 0.606 | III (> 10 cm) vs II (4 - 10 cm) | 0.918 | 0.773 | 1.091 | 0.332 |
|  |  | III (> 10 cm) vs I (< 4 cm) | 0.910 | 0.721 | 1.149 | 0.428 |
|  |  | II (4 - 10 cm) vs I (< 4 cm) | 0.991 | 0.824 | 1.193 | 0.924 |

**Suppl. Table 8:** Multivariable analysis results for pain requiring treatment, including odds ratio estimated with corresponding 95% confidence intervals.

* Logarithmic transformatio
